# Numerical Simulation of Airborne Disease Spread in Cage-Free Hen Housing with Multiple Ventilation Options

**DOI:** 10.3390/ani12121516

**Published:** 2022-06-10

**Authors:** Long Chen, Eileen E. Fabian-Wheeler, John M. Cimbala, Daniel Hofstetter, Paul Patterson

**Affiliations:** 1 Department of Agricultural and Biological Engineering, The Pennsylvania State University, University Park, PA 16802, USA; d.hofstetter@ufl.edu; 2 Tianjin Academy of Agricultural Sciences, Tianjin 300384, China; 3 Department of Mechanical Engineering, The Pennsylvania State University, University Park, PA 16802, USA; jmc6@psu.edu; 4 Department of Agricultural and Biological Engineering, University of Florida, Gainesville, FL 32611, USA; 5 Department of Animal Science, The Pennsylvania State University, University Park, PA 16802, USA; php1@psu.edu

**Keywords:** cage-free hen housing, ventilation system, computational fluid dynamics, airborne disease, numerical simulation

## Abstract

**Simple Summary:**

Airborne diseases, such as highly pathogenic avian influenza, are among the deadliest threats to the egg industry and can easily cause devastating losses of poultry when severe outbreak events occur. During the ongoing transition to cage-free production, uncertainty regarding ventilation designs for cage-free facilities also exposes vulnerability with respect to disease control within facilities. To address ventilation system design and the capability of restraining internal airborne disease spread, this study was conducted to model and compare indoor airborne virus dispersal for a commercial cage-free hen house within four different ventilation schemes. A one-eighth length, full-scale, floor-raised hen house with commercial bird density was modeled to simulate the environmental conditions and disease spread under steady-state conditions inside the barn during cold weather. Analyses of the dispersion of virus particles coupled with airflow patterns were performed by visualizing contours of virus particles and air velocities at critical locations. In addition, the virus mass fraction at bird level was of particular interest when comparing and evaluating the performance of various ventilation schemes. The simulation results demonstrated that the internal dispersion of airborne virus particles was determined by indoor airflow patterns and implied the role of ventilation configuration in reducing disease spread in a poultry barn. Furthermore, valuable insights are provided for further investigations of ventilation options for cage-free hen housing.

**Abstract:**

The current ventilation designs of poultry barns have been present deficiencies with respect to the capacity to protect against disease exposure, especially during epidemic events. An evolution of ventilation options is needed in the egg industry to keep pace with the advancing transition to cage-free production. In this study, we analyzed the performances of four ventilation schemes for constraining airborne disease spread in a commercial cage-free hen house using computational fluid dynamics (CFD) modeling. In total, four three-dimensional models were developed to compare a standard ventilation configuration (top-wall inlet sidewall exhaust, TISE) with three alternative designs, all with mid-wall inlet and a central vertical exhaust. A one-eighth scale commercial floor-raised hen house with 2365 hens served as the model. Each ventilation configuration simulated airflow and surrogate airborne virus particle spread, assuming the initial virus was introduced from upwind inlets. Simulation outputs predicted the MICE and MIAE models maintained a reduced average bird level at 47% and 24%, respectively, of the standard TISE model, although the MIRE model predicted comparable virus mass fraction levels with TISE. These numerical differences unveiled the critical role of centrally located vertical exhaust in removing contaminated, virus-laden air from the birds housing environment. Moreover, the auxiliary attic space in the MIAE model was beneficial for keeping virus particles above the bird-occupied floor area.

## 1. Introduction

The shift to cage-free egg production is the most significant evolution in poultry facilities that has been faced in decades [1]. Aviary systems, convertible cages, and floor housing systems are commonly called cage-free poultry barns, despite no unified guidelines for “cage-free” housing [2,3,4]. The number of hens and sizes of newly constructed hen houses have increased significantly, driven largely by competitive markets and narrow profit margins, which escalate the pressure on the design of cage-free housing systems [5]. Thus, more investments and efforts are needed to maintain a satisfactory indoor environment and balance the demand of production with animal welfare in large-scale, commercial, cage-free hen houses. In addition, decreasing the risk of airborne disease spread is crucial to the economic viability of cage-free barns, as well as ensuring bird welfare [6,7]. However, current cage-free ventilation designs present some challenges in providing uniform, fresh, and comfortable air, exposing deficiencies associated with coping with disease outbreaks. Therefore, current ventilation designs need to evolve concurrently to keep pace with the advancements of poultry facilities during the transition to cage-free egg production.

Moreover, the capacity of ventilation systems to constrain airborne disease spread is paramount during epidemic outbreaks. Airborne or aerosol transmission is a typical route of infectious disease, which occurs when birds inhale fine particles or aerosols that contain contagious pathogens. Airborne transmission can take place at greater distances because diseases can be dispersed through airstreams from outdoor to indoor environments and from bird to bird. Such transmission can be worse if the pathogen-laden air is easily transported through most bird-occupied areas prior to being exhausted, as conventional ventilation schemes are designed to maintain indoor animal comfort and may neglect the importance of disease control. During the spring of 2022, an ongoing outbreak of highly pathogenic avian influenza (HPAI) was reported throughout the United States, leading to the loss of millions of birds to date at, representing the worst loss since 2015 [8,9]. The H5N2 HPAI outbreak in late 2014 to 2015 affected 232 poultry barns and resulted in the loss of nearly 50 million birds in the USA [6]. Epidemiological investigations revealed that some of the transmission likely occurred via aerosolized virus airflows within a single poultry barn and even between closely spaced barns, which prompted the rapid spread of the HPAI virus [10,11]. Therefore, a well-designed ventilation scheme should not only maintain good indoor air quality but also be capable of preventing the uptake and spread of contagious contaminants within the house.

Evaluating ventilation performance to constrain airborne disease dispersal in a commercial hen house is challenging and laborious, with the infeasibility of exposing birds to pathogenic contaminants. Instead, a mathematical simulation methodology is preferred as an alternative approach to field measurements [8]. Computational fluid dynamics (CFD) models have been widely used to study fluid flow in numerous application scenarios [12,13]. Agricultural engineers have embraced CFD modeling as a sophisticated numerical approach to simulate airflow in various agricultural production facilities to address environmental problems with specific aims, as CFD modeling is highly objective-oriented [14]. In particularly, using CFD simulations to evaluate agricultural ventilation designs is economical in terms of time and capital costs [15,16,17,18].

The need to address problems associated with airborne disease dispersion in poultry houses has encouraged researchers to use CFD simulations to assess ventilation designs. In a previous study, we modeled the mass flux profile of virus particles in two different hen housing ventilation schemes that featured opposite airflow directions in two dimensions [19]. In another study, Tong et al. (2019) evaluated the performance of an upward airflow displacement ventilation system in comparison with a typical tunnel ventilation system via CFD modeling and assessed air-exchange effectiveness, thermal environment, and the capacity to inhibit airborne disease dispersion [20]. To study the spread of HPAI viruses originating inside poultry houses and evaluate the spatial impact on the surrounding environment, researchers utilized CFD models in a three-dimensional topography that was coupled with a geographic information system (GIS) and varying weather conditions [21]. The simulation outputs were deployed as background data to construct HPAI spread networks, which were analyzed using centrality methods to identify suspected barns, as well as highly vulnerable barns, to prevent further spreading by tracing the infection routes [22]. However, most of previous CFD models were based on conventional poultry facilities; few studies have been conducted to investigate the ventilation design for emerging cage-free hen facilities with an emphasis on airborne disease control.

An existing commercial cage-free hen house was modeled at full-scale in three dimension, and four ventilation configurations were simulated using CFD to evaluate ventilation system performance with respect to reducing indoor virus particle transmission at the bird level. Three-dimensional CFD models were developed for a barn with a standard ventilation configuration with realistic hen numbers compared to three alternative designs. Numerical simulations of airflows, along with the dispersion of virus particles during cold weather, were conducted to characterize the dispersal patterns of viruses by calculating their mass fraction profiles. The goal was to compare the performance of each ventilation design in controlling airborne disease spread in typical cage-free hen housings with an emphasis on analyzing the influence at the bird level in order to provide practical recommendations for future refinement of cage-free ventilation options.

## 2. Materials and Methods

Three-dimensional models and pertinent simulations were conducted by deploying commercial CFD code FLUENT (ANSYS v19.1, Canonsburg, PA, USA) [23]. Computations for this research were performed on the Roar supercomputer of the Pennsylvania State University’s Institute for Computational and Data Sciences. The standard k-ε turbulence model coupled with enhanced wall functions [13,24] was adopted for numerical simulation of airflow and disease dispersal using steady-state calculations on the basis of previous investigations [19,25,26].

### 2.1. Floor-Raised Hen House and Ventilation Schemes

A floor-raised hen house in Lititz, Pennsylvania, was selected as the commercial house for CFD modeling. The house is 162.15 m (532 ft) long and 13.72 m (45 ft) wide, with a capacity of almost 20,000 birds. Details of the study hen house can be found in our previous publications [3,4,27].

**Figure 1:**
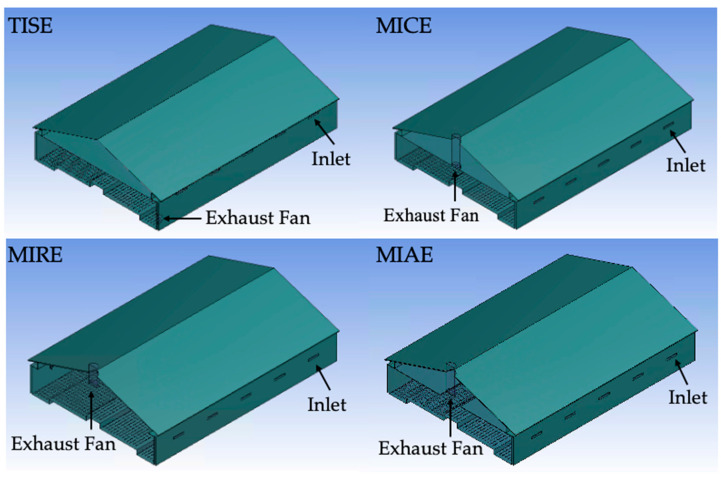
Diagrams of the study hen house with four ventilation configurations (the standard, TISE, and three alternative designs: MICE, MIRE, and MIAE) and annotations indicating the position of inlets and the exhaust fan.

A total of four ventilation schemes were modeled, including the standard ventilation system currently in use, as well as three alternative designs (Figure 1). The current ventilation scheme (a standard in North American poultry facilities) for cold and mild weather creates indoor negative pressure driven by the exhaust fans on one sidewall, forcing fresh air into the barn through inlets beneath the eaves, which is a standard ventilation system used in North American poultry facilities and referred to as TISE (top-wall inlet sidewall exhaust) [28,29,30].

Three alternative ventilation systems, namely, MICE (mid-wall inlet ceiling exhaust), MIRE (mid-wall inlet ridge exhaust), and MIAE (mid-wall inlet attic exhaust) had identical inlets positioned 1.5 m (59 in.) above the ground that worked with opening baffles and wall plates at the top, yet with different exhaust placement and ceiling features [4]. The mid-wall inlet and centrally located vertical exhaust is more typical in European designs. The MICE exhaust fan was positioned at the middle of the ceiling with a 2.9 m (112.5 in.) fan chute attachment. No ceiling was utilized for the MIRE, and the exhaust fan was positioned 3.3 m (131 in.) above the centrally located nest boxes, with a 1.6 m (64.5 in.) long duct attached from the ridge. To create an air treatment option for air prior to exhausting, the MIAE hybrid ceiling design was included with a 2.3 m (90 in.) wide open area along the centerline of the ceiling, creating an attic area, where the exhaust fan was positioned in the duct connected to the middle of roof ridge [31].

### 2.2. Procedure for CFD Modeling

#### 2.2.1. Preconditions and Assumptions

One-eighth of the study hen house was modeled to take advantage of construction symmetry and save computational resources. The modeled hen house was assumed to have complete insulation, which was not realistic but simplified the primary heat loss during cold weather conditions from exhausting of warm indoor air via the convection ventilation system. Ten inlets with an identical opening size of 3.81 cm (1.5 in.) were modeled for the hen house and preconditioned to provide adequate airflow at a pertinent static pressure difference [32]. Only the half an exhaust fan was necessary to maintain a desired ventilation rate in the model. Another critical assumption was that all the hens were uniformly distributed inside the barn with constant body temperatures. The airborne viruses were presumed as extremely small particles or aerosols, the dispersion behavior of which was considered equivalent to the spread of a contaminant gas, assuming no phase change [19]. In addition, the cold weather simulation was fixed at 0 °C (32 °F), and the wind was assumed to blow horizontally at a constant speed of 2 m/s (393.7 ft/min). All gaseous materials were defined as incompressible ideal gases, and the humidity variation within the barn was not addressed in the scope of this study. The surface of an individual hen was presumed to be maintained at a constant 42 °C (107.6 °F) [19,33].

#### 2.2.2. Computational Domain

The computational domain of each model involved the barn and ambient air for the sake of analyzing indoor and outdoor airflows. A uniform domain size of 128.2 m × 20.3 m × 24.4 m (420.6 ft × 66.5 ft × 80 ft) was established for each of the four models. To reduce the end-wall effects, only a central section representing one-eighth of the study hen house was modeled with realistic dimensions and representative ventilation features. The designated extra downwind space in the domain was designated to minimize the reverse flows at the boundaries during numerical simulation [28].

#### 2.2.3. Modeling Individual Hens

To approach realistic simulations and assess disease spread at the hen level, 2365 individual hens were modeled to represent approximately one-eighth of the whole flock size. An individual hen model was created using a simplified torso–head–tail shape with a surface area of 0.11 m^2^ (1.18 ft^2^) to stand for an average body weight of 1.59 kg (3.5 lb.) [2,3,34]. A uniform area occupied per hen model was calculated according to the actual stocking density [3,4].

#### 2.2.4. Boundary Conditions

The CFD simulations adopted six types of boundary conditions or cell zones. The “wall” boundary conditions included the ground, the top surface of the computational domain, hen model surfaces, and all the components of the hen house, such as ceiling, roof, nesting area, etc., which were all set as non-slip walls, except for the top surface of the domain, which was defined as a zero-shear stress wall [2,3,4,19]. “Symmetry” boundary conditions were assigned to the front and the back surfaces of the domain to stand for internal faces. The left end of the entire domain was defined as “velocity inlet” with a constant magnitude of 2.0 m/s (393.7 ft/min) horizontally to the right. A “pressure outlet” with a specified gage pressure value of 0 Pa was assigned to the right end of the domain, where the flow exited to the atmosphere. Faces perpendicular to the initial wind direction in each inlet and the exhaust fan, were defined as boundary conditions of “interior”. The body of the exhaust fan was defined as a “3D fan zone” to simulate the driving force from an axial fan by applying a distributed momentum source with a constant pressure jump value [35]. To ensure that all the models had similar ventilation rates, the pressure jump of the TISE, MICE, MIRE, and MIAE models were individually adjusted to 18 Pa, 20 Pa, 16 Pa, and 15 Pa, respectively.

#### 2.2.5. Modeling Airborne Disease Dispersal

To simulate the spread of airborne disease, gaseous ammonia was selected as a surrogate contaminant species, as the dispersal behavior of virus particles can be analogous to the diffusion of a tracer gas [2]. The diffusion energy source was enabled for the species model of the ammonia–air mixture. To describe the dispersal of an airborne virus particles, a species transport model without reactions was adopted. Thereby, 100% (a mass fraction value of 1) ammonia was introduced at the surfaces of five upwind inlets to simulate airborne virus particles in the incoming ventilation air and the spread of disease inside the study hen house.

#### 2.2.6. Meshing and Solver Settings

ANSYS meshing was deployed to discretize the computational domain [22]. Skewness was used to assess meshing quality before launching the CFD simulation. A mesh-convergence study was conducted using a uniform grid convergence index (GCI) based on a mesh refinement error estimator derived from the generalized Richardson extrapolation [4,36]. The final mesh of the TISE, MICE, MIRE, and MIAE models included 12.1, 12.4, 12.3, and 12.3 million cells, respectively. Details of the mesh-convergence study can be found in the previous article [4].

A pressure-based solver was used to conduct all simulations. Governing equations were discretized using a second-order scheme, and a first-order scheme was adopted to calculate turbulent kinetic energy and turbulent dissipation rate.

The convergence criteria were not met until both the monitoring variable at selected points and the residual values were stabilized, satisfying the scale threshold. For all simulations, 2500 iterations were applied to ensure all the meshing files were generated with a similar size.

### 2.3. Post-Processing of Simulation Data

#### 2.3.1. Data Visualization

Three parallel cross-sectional reference planes were created to characterize simulation outputs, which represented locations in a barn with inlets (CP-I), no ventilation features (CP-N), and an exhaust fan (CP-F), respectively [3]. A row of hen models was crossed by each reference plane to analyze disease dispersal at the bird level. CP-N-type cross sections occupied 69% of the indoor space, according to the ventilation features, which was roughly two thirds of the entire barn.

In addition, a transversal reference plane TP-I was created to visualize the incoming air and viruses, which was parallel to the ground through the middle of individual inlets in each model. The TP-I was set to 2.6 m (8.53 ft) above the floor in the TISE model, whereas the TP-I in the MICE, MIRE, and MIAE models was set to a height of 1.6 m (5.25 ft).

Five bird-occupied zones were created to capture the simulation data at the bird level. Each zone represented conditions at different locations within a cross-sectional reference plane covering all the floor area [2,3,4]. Each zone was 0.25 m (10 in.) tall to represent the height of the hen air space and varied in width from 1.78 m (5.83 ft) to 3.94 m (12.9 ft) depending on its use as nest-box area (in center), raised feeder and drinker spaces, or litter scratching areas along each sidewall. Finally, a comparison of disease concentrations with spatial heterogeneity was performed by analyzing data from different zones in a specific plane.

Simulation results of environmental parameters of interest were previously analyzed by creating contours of airflow vectors, temperature distribution, and static pressure difference [3,4,29,30]. In this study, the dispersal of surrogate airborne viruses was investigated, coupled with airflow patterns within multiple ventilation schemes, to compare the performance of each ventilation system in containing indoor virus spread. To visualize the spread of viruses inside the hen house, simulation results of mass fractions were employed to create rendered volumes and contours to analyze the virus dispersal within each model. The scale of the color legend was set between 0 and 0.03 to achieve finer resolution.

#### 2.3.2. Statistical Analysis

In addition to characterizing the virus spread from a perspective of the entire barn, point-to-point data were exported from five bird-occupied zones to compare and evaluate the potential threat at the bird level with the aid of statistical analysis. To quantitatively analyze the performance of different ventilation schemes in controlling the spread of airborne disease, environmental parameters and mass fraction data of ammonia were exported from each cross-sectional reference plane. All the simulation data points were treated as repeated measurements that were fit to a mixed-effects model to verify whether altering ventilation schemes and locations (zones) had statistically significant effects on the results of interest. Analysis of variance (ANOVA) and Tukey tests were employed to test pertinent hypotheses and perform multiple comparisons using R v4.1.2 [37,38].

## 3. Results

### 3.1. Simulated Indoor Airflow within Four Ventilation Schemes

**Figure 2:**
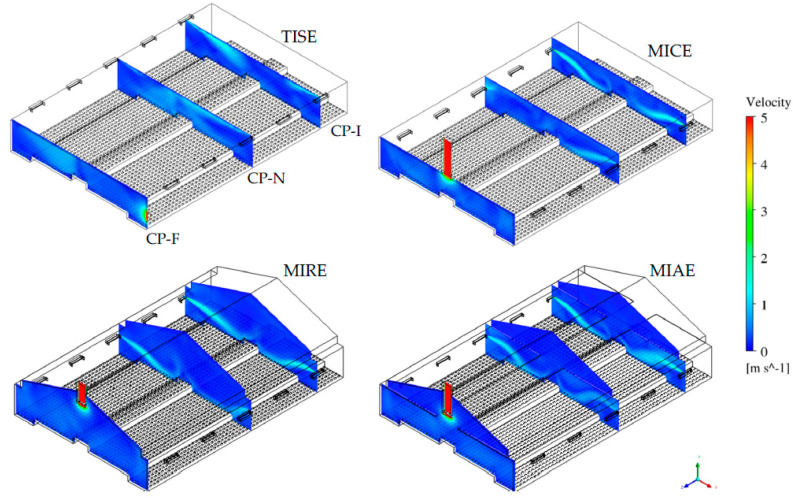
Isometric view of the four hen house ventilation schemes for the TISE, MICE, MIRE, and MIAE models with three cross-sectional reference planes (CP-I, CP-N, and CP-F) depicting airflow magnitudes at critical locations. The color scale was set between 0 and 5 m/s, and the hens appear as dots on the floor in this view.

The total ventilation rate of the study hen house within each ventilation scheme was 1.97 m^3^/s (4174 ft^3^/min), 1.93 m^3^/s (4089 ft^3^/min), 1.96 m^3^/s (4153 ft^3^/min), and 1.91 m^3^/s (4047 ft3/min) based on the simulation output of the TISE, MICE, MIAE, and MIAE models. In addition, the internal volume of the barn varied among the four models, namely 639.42 m^3^ (22,581 ft^3^), 639.36 m^3^ (22,579 ft^3^), 1058.25 m^3^ (37,372 ft^3^), and 1052.67 m^3^ (37,175 ft^3^) for the TISE, MICE, MIRE, and MIAE models, respectively. The TISE and MICE barns had an identical internal volume with a flat ceiling, whereas the MIRE and MIAE barns had greater interior volume either without a ceiling or with an attic space (partial ceiling). Air velocity contours at three cross-sectional reference planes are presented as geometric illustrations of the hen house with four ventilation schemes to visualize the indoor air patterns in each case (Figure 2).

At CP-I, rapid incoming air was observed at inlets, despite the heterogeneity of ventilation designs. The left-side incoming air jets tended to have longer trajectories compared to those from the right side due to upwind influence. The standard TISE model had inlets at the top of both sidewalls close to the flat ceiling, along which incoming air jets can move. Due to the lower sidewall location of inlets among the three alternative models, the incoming air jets tended to decline towards the bird-occupied area, resulting in obvious regions of fast airflows at the location near the central, raised nest-boxes.

Strong internal air movements at CP-N were observed in all models due to the impact of adjacent inlets. However, the airflow patterns varied dramatically within each ventilation scheme. Air velocity contours suggested more active movements overall in the TISE model compared to the three alternatives; the most vigorous airflows were presented in the middle of TISE model, with some fast airflows accumulating close to the ceiling on the right side. MIRE and MIAE had similar fast airflows on the left-side, with similar shapes of upwind air jets at CP-I. The rapid airflows on the right-side appeared to be slightly weaker in terms of smaller magnitudes and shorter trajectories. In contrast to observations in the MIRE and MIAE models, robust airflows in the MICE model were unveiled on the downwind right side, whereas a rather small, light blue area was noticed in the upper left portion, indicating some fast airflows.

Airflows at CP-F were influenced by the exhaust fan, yet differed significantly between the standard TISE model and the three alternatives because of the different location of the fan. In general, almost no obvious fast airflow regions in the three alternative models were observed in that plane, except for the “dome” area close to the fan, which matches observations in actual hen houses [31,38]. Nevertheless, air movements tended to be a bit more robust in the TISE model, with a light blue region in the left portion, in addition to the fan area.

### 3.2. Simulated Dispersal of Airborne Viruses

#### 3.2.1. Overall Indoor Dispersal of Airborne Viruses within Four Models

**Figure 3:**
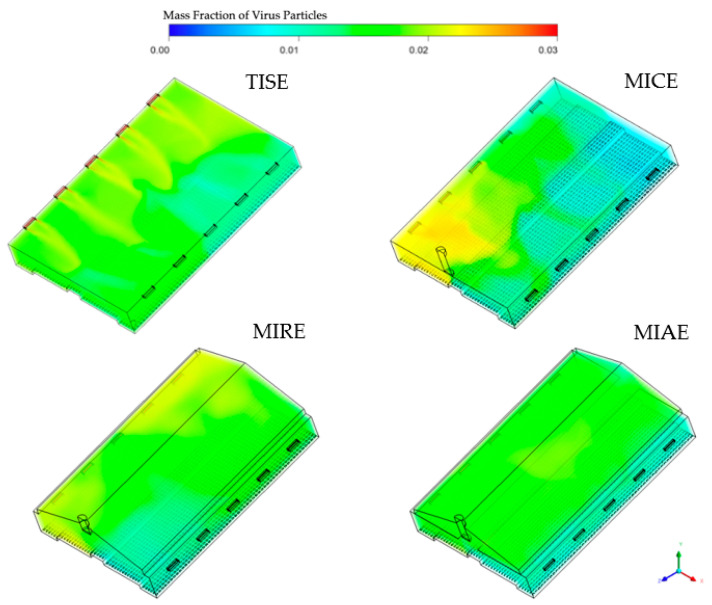
Three-dimensional illustration of four models representing ventilation schemes rendered with corresponding simulation results of indoor mass fractions of airborne viruses. Note that the transparency is inversely proportional to the magnitude of the mass fraction.

Simulation results of mass fractions of virus inside the barns (Figure 3) suggested heterogenous patterns within the four models, indicating an impact of the ventilation configuration on the airborne dispersal of virus. In general, most of the indoor space was maintained between 0.01 and 0.02 e.g., mostly represented by light blue and green colors. 

The TISE, MICE, and MIRE alternative ventilation models had overall lower concentrations of virus on the downwind right side of the barn compared to the left side, which was reasonable, as the virus was introduced from upwind inlets. Because inlets in the TISE model were located close to the ceiling, high concentrations of virus greater than 0.02 were observed at the edges of inlets. In the MICE model, accumulations of virus particles were located in the left portion close to the exhaust fan, with some concentrations approaching 0.025. Outstanding yellowish areas appeared in the left corner close to the far end in the MIRE model, with no obvious reddish or orange (high-concentration) regions. However, slight yellowish and green areas were observed in the upper central portion of the barn in the MIAE model, indicating moderate accumulations of virus in the attic area.

#### 3.2.2. Transmission of Viruses at Inlets

**Figure 4:**
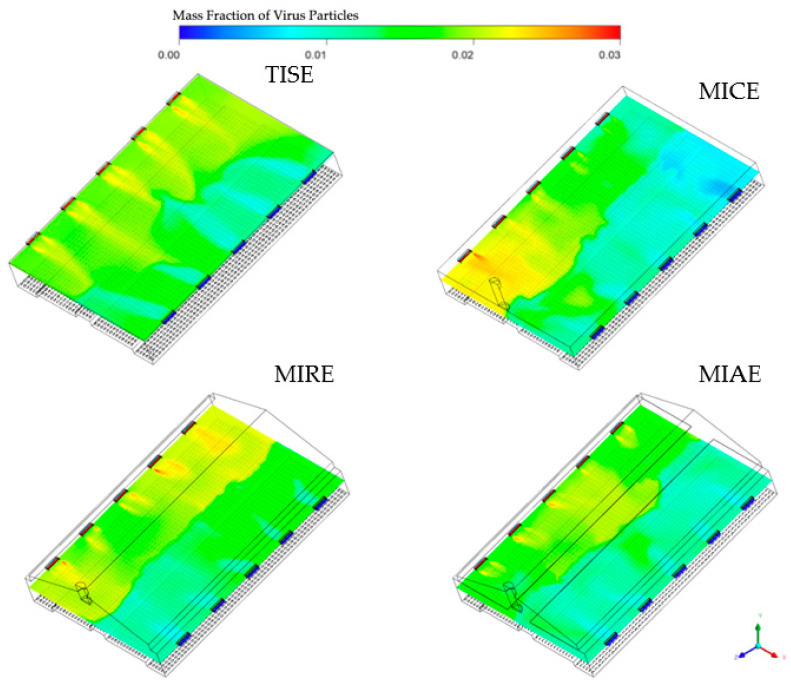
Contours of virus mass fractions at TP-I in the four models. Simulated virus particles were introduced from upwind side inlets (left).

Transversal planes (TP-I) of virus particles within each model (Figure 4) showed some red on the left-side inlets, indicating an extremely high concentration of viruses, which is consistent with the pre-defined boundary conditions, whereas the air-laden inlets and their spaces on the other side showed dark blue color, representing no virus. 

Incoming streams of simulated virus particles resulted in distinct color sections of contamination in all four models, with boundaries in the middle of the plane, especially in the MIRE model. Due to longer trajectories of incoming air jets in the TISE model, streams containing high concentrations of viruses were also longer than those of the other three models. Concentrations of virus particles tended to be higher from the inlets that were close to the exhaust fan in the MICE model, in contrasted to observations in the MIRE model. The largest orange-reddish area in the MIRE model was observed near the inlet at the end farthest away from the exhaust fan. Incoming streams of viruses in the MIAE model were relatively uniform, with a short length of orange-reddish trajectories, whereas a yellow region was formed in the central part, indicating moderate accumulations of virus particles near the attic entrance.

#### 3.2.3. Disease Spread at Cross-Sectional Reference Planes

**Figure 5:**
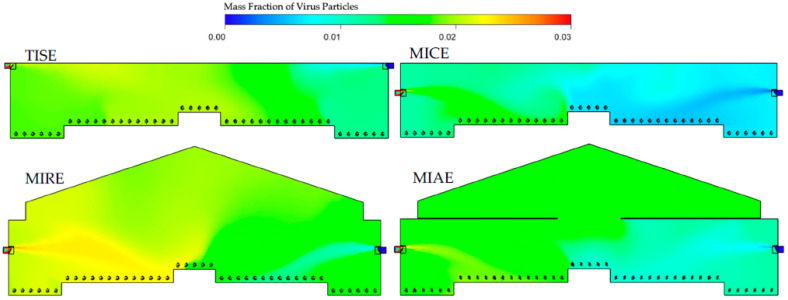
Indoor mass fraction contours of virus particles in the four models (TISE, MICE, MIRE, and MIAE) at the cross-sectional reference plane of CP-I. Individual modeled hens are shown at floor level.

**Figure 6:**
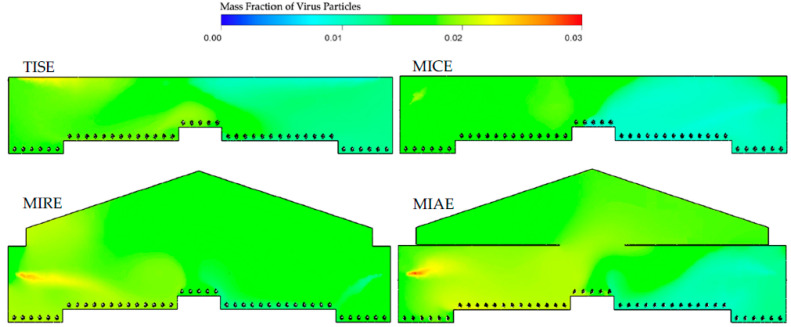
Indoor mass fraction contours of virus particles in the four models (TISE, MICE, MIRE, and MIAE) at the cross-sectional reference plane, CP-N.

**Figure 7:**
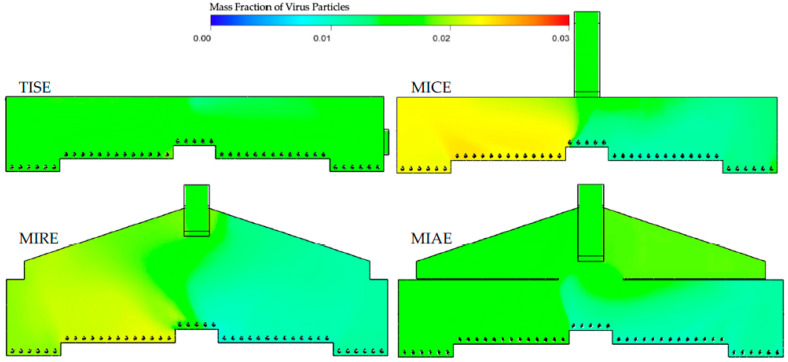
Indoor mass fraction contours of virus particles in four models (TISE, MICE, MIRE, and MIAE) at the cross-sectional reference plane, CP-F.

The dispersal behavior of virus particles differed at three cross-sectional reference planes and was influenced by the ventilation features of inlet and fan location. The contours of mass fraction to visualize the distribution of virus concentrations are shown in a steady state in Figure 5, Figure 6 and Figure 7.

At CP-I, the influence of the inlets suggested significant effects on the internal air circulation, as well as virus spread (Figure 5). The distribution of virus particles varied within different ventilation systems. The standard TISE model presented a level of viruses between 0.020 and 0.022 from the upper left to the central portion of the barn, with an average virus mass fraction of 0.017 in this plane. In addition, the range of indoor virus mass fractions in the TISE model at CP-I was 76% to 79%, which is lower than the other three models, indicating more uniform internal air mixing. Fresh air introductions with a low mass fraction of virus and a large blue region were observed on the right-side inlet in the MICE model. However, the MIRE model had an obvious yellow region of higher viral load at the left inlet extending to the bird-occupied areas close to the nest boxes, whereas the fresh air jet from the right-side inlet was not as robust as in the other models. The attic area in the MIAE model was uniformly green, with lower viral load likely as a result of thorough mixing of virus particles with internal air circulation. Additionally, the injection of viruses and fresh air was observed separately from the two sides of the barn, although neither incoming trajectory was obvious in the MIAE model. For the three alternative designs, the average mass fractions of virus particles were 0.010, 0.018, and 0.013 for MICE, MIRE, and MIAE, respectively, based on simulation outputs at CP-I.

Differences in virus mass fractions were observed at CP-N between the left and right sides of the barn in all models, although no ventilation features are part of this plane (Figure 6). Average mass fractions of viruses in each model were quite close, namely 0.016, 0.013, 0.017, and 0.016 for the TISE, MICE, MIRE, and MIAE models, respectively. A yellow region near the ceiling was observed in the upper-left corner in the TISE model. Similarly, a small region with high mass fractions of viruses was observed close to the left sidewall in the MICE model. Although no inlets were present in this plane, the orange and reddish regions were influenced by nearby inlets on the left side in the MIRE and MIAE models, projecting towards bird-occupied regions. In addition, light blue regions with low virus mass fractions below 0.01 were noticed at the bird level in the right portion of the MICE and MIAE barns. In the TISE model, a relatively small, light blue region was observed close to the ceiling in the right portion with incoming fresh air, yet no such obvious regions with low level of viruses were detected in the MIRE model.

The mass fraction contours at the CP-F plane in the TISE model showed more uniformity compared to the other three models (Figure 7). No visible yellowish or orange color was observed in the region close to the TISE fan. The average TISE mass fraction was 0.016, which is almost the same as that in the MIRE model. In addition, MIAE had the lowest average virus level of 0.014, with a light blue region in the right portion of barn, excluding the attic area. However, both the MICE and MIRE models had regions with high levels of virus on the left side, particularly at the bird level in the MICE model. In all three alternative models, relatively lower levels of virus were detected on the right side of the barn, as most of the contaminants were exhausted from the vertical fan to the atmosphere in the center of the barn cross section. Nevertheless, no significant increase in virus concentrations was observed inside the fan or fan chute.

### 3.3. Virus Dispersion at Bird Level

Virus mass fraction data at bird level were captured and exported for quantitative analysis to compare the internal airborne disease within each ventilation scheme. Simulation data from the five bird-occupied zones were analyzed separately at CP-I, CP-N, and CP-F reference planes to compare virus levels from each ventilation model.

The effects of key factors and their interactions were statistically significant in terms of mass fractions of virus based on the outputs of ANOVA analyses at three separate reference planes (Table 1). The number of data points exported from the CP-I, CP-N, and CP-F planes was 35,340, 34,986, and 34,178, respectively. Statistical analyses demonstrated that the ventilation model and locations of the zone had a critical impact on the virus level at the bird level.

Subsequent Tukey’s tests were performed to test the statistical significance of pair-wise differences of the means of mass fraction data from each plane. The results of statistical analyses demonstrated that most of the differences between pairwise comparisons were statistically significant due to the large scale of data points from a single zone (Table 2), although the actual values of the differences of mass fractions were rather small.

**Figure 8:**
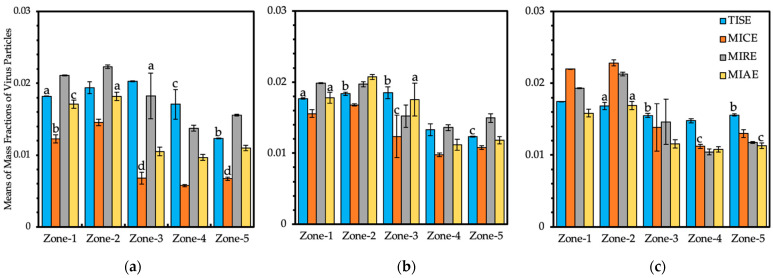
Mass fraction means of virus particles from five bird-occupied zones at three cross-sectional planes, (**a**) CP-I, (**b**) CP-N, and (**c**) CP-F, in four models: TISE, MICE, MIRE, and MIAE. Annotations with the same letters indicate **NO** statistical significances were found for the differences between the comparisons at a 95% family-wise confidence level. Error bars indicate standard deviations of simulation data points in each zone.

Bar graphs were created for simulation results of mass fractions from five bird-occupied zones in the same plane with annotated statistical significance (Figure 8). Due to the influences of inlets at CP-I, higher average mass fractions were observed in the left portion of Zone-1 and Zone-2 than those of Zone-4 and Zone-5, which were consistent in all four models (Figure 8a). In addition, the highest average mass fractions of virus were observed in Zone-2 in the three alternative models, which was different than the standard TISE model, which presented the highest mean fraction value in Zone-3. The TISE model directed the inlet air jet further across the top of the hen house, falling into the bird-occupied area at the center of the building, whereas the three alternative model mid-wall inlets directed air toward the bird level between the center and sidewall of the house (Figure 2 and Figure 5). In the central portion, Zone-3, the TISE model had the highest mean of virus level, which was 11% higher than the second highest value in this zone, which was observed in the MIRE model. In fact, the MIRE model presented the highest average virus level at CP-I according to data from all five zones, which is consistent with the observations from its mass fraction contours (Figure 5). In Zone-4, the mean value of viruses was 0.0058 in the MICE model, which was the lowest virus level for a single zone from all three planes, on average. Among all four models, MICE presented the lowest virus level at CP-I, with an average value of 0.0092, which was approximately 31% lower than MIAE, 47% lower than TISE, and 49% lower than MIRE. In addition, the MIAE model maintained the second lowest virus level of 0.0133, which was 24% lower than that in the TISE and 27% lower than that in the MIRE model.

Mass fraction data from five zones at CP-N (Figure 8b) suggested some similar patterns with outputs of CP-I. Means of mass fractions in four models presented steep declines in Zone-4 and Zone-5 compared to the data from Zones 1–3. The TISE model had its highest average virus level in Zone-2 and Zone-3, and no statistically significant differences were found between those two zones. The highest mean of mass fractions in the MIRE model was observed in Zone-1, although the second highest mean was extremely close, in Zone-2. For the MICE and MIAE models, the highest mean of mass fractions in a single zone was found in Zone-2. Again, the MICE model had the lowest virus level (0.0131) on average, taking account of all five zones, which was 17% lower than that of the MIAE model, 19% lower than that of the TISE model, and 28% lower than that of the MIRE model.

Scenarios at CP-F (Figure 8c) presented different results compared with the previous two planes, which indicated the impact of the exhaust fan on indoor virus spread. The average virus level from each zone in the TISE model was quite close, ranging from 0.0148 to 0.0174. The three alternative ventilation systems all had their maximum mass fractions of viruses in Zone-2. Interestingly, the mean virus level of the MICE model in Zone-2 was 0.0228, which was the highest value of all the exported data from a single zone in the three planes. Additionally, the MICE model presented the maximum mean of virus mass fractions in CP-F in the entire five zones, with a value of 0.0166, which was 3% higher than that of the TISE model, 7% higher than that of the MIRE model, and 25% higher than that of the MIAE model.

The large error bars in the MICE, MIRE, and MIAE models in Zone-3 indicate dynamic virus concentrations at the bird level close to the nest-box region. However, the variation of mass fractions was not obvious in Zone-3 within the standard ventilation system of the TISE model compared to the three ventilation alternatives, implying a relatively steady air movement in that region.

## 4. Discussion

Previous studies have demonstrated that the performances of these three alternative ventilation schemes represented by the MICE, MIRE, and MIAE models are capable of providing indoor environmental conditions (temperature, air speed, and static pressure) comparable with those of the industry-standard TISE ventilation scheme [3,4,29,30,31]. Although field validation studies were not included, the simulation results appear rational and agree with the observations in commercial poultry houses, as well as findings of other relevant studies [32,39,40]. The average air speed at the bird level for the TISE model was predicted to be 0.26 m/s (51 ft/min), which is in agreement with the measured data of a broiler poultry barn (0.25 m/s) with a similar house size and bird population during winter [18], despite the slight differences in ventilation schemes between the two poultry barns. In addition, our simulation output of the TISE model had an average temperature of 21.28 °C at the bird level, which is very close to the measured temperature of 21.14 °C reported in a study by the same group [18]. In the present study, comparisons of the ability to constrain airborne disease spread were conducted between the TISE and three alternative models, which were facilitated by steady-state simulations of dispersal of surrogate virus particles in the study hen house. Although no suitable studies are available for comparison, the internal traveling patterns of airborne viruses were generally consistent with our previous findings for a conventional caged-layer house using two-dimensional simulation [19].

Because viruses were initially injected through the upwind (left-side) inlets, all four models showed a consistent general tendency wherein the left portion of the barn had higher mass fractions of virus than the right portion. Analyses of virus mass fractions at three cross-sectional references planes revealed similar or even lower virus levels, on average, within the three alternative models compared with the standard TISE model. The MICE model had an indoor volume identical to that of the TISE model, although the average virus levels at the bird level were 47% and 19% lower in the CP-I and CP-N planes, respectively, and were only 3.4% higher in the CP-F plane. The average virus level in the MIAE model was just 1.4% higher than that in the TISE model at CP-N yet was 24% and 17% lower than the TISE model in the CP-I and CP-F planes, respectively, at the bird level. According to the analyses of simulation results from five bird-occupied zones in each reference plane, the MICE and MIAE models exhibited more competitive performances with respect to maintaining low concentrations of viruses relative to the standard TISE model, whereas the virus level in the MIRE model was slightly higher than that in the TISE model at the bird level (Table 2 and Figure 8).

Hence, the MICE and MIAE models have advantages in terms of internal air circulation over the TISE model from the perspective of maintaining lower levels of airborne particles under the same simulation conditions. In fact, the MICE model represents a ventilation configuration that is widely used in Europe, forming an overall bottom-to-top airflow path inside the barn, which might be a more efficient design to exhaust the airborne contaminants that were absorbed from the upwind atmosphere [20,41]. By visualizing transverse contours of virus mass fractions in the MICE model, we noticed accumulations of virus surrounding the exhaust fan in the left portion of the barn (Figure 4). This is a sign of numerous air circulations that eventually moved virus-laden air to the fan area. Due to the incoming fresh air and buoyancy, airflows containing viruses hardly had a chance to reach the right portion of the barn, especially the lower bird-occupied area, which might explain the observations of considerable green and blue regions in the MICE model (Figure 5, Figure 6 and Figure 7).

In contrast, such internal air circulations may not be as efficient as in the MIRE and MIAE models due to the different ventilation configurations. However, the partial ceiling of in the MIAE model played a critical role in containing the virus-laden air inside the attic by blocking vertical air movements in the process of internal air circulation. As a result, the average virus level in the MIAE model was the second lowest at the bird level at CP-I and CP-N and was the lowest at CP-F among all models, as a majority of viruses were trapped in the recirculating zone in the attic space. This evidence warrants further exploration of the use of an attic area for pretreatment of exhaust air. Without any ceiling, vertical air movement took place frequently in the case of the MIRE model, which enabled viruses to be transmitted thoroughly in the left portion of the barn [4]. Due to a larger indoor space, the air changes per hour (ACH) from the ventilation rate of the MIRE model may have allowed more significant accumulation of virus compared to the MICE model. The mechanical ventilation rate was determined based on bird needs for air quality and not equivalent ACH. Therefore, accumulations of viruses were realized at the far end in the contours of the MIRE model barn (Figure 4 and Figure 5).

Herein, indoor dispersal of virus particles spread was investigated, assuming the virus was initially introduced through upwind inlets with preconditions of constant wind (speed and direction). The evaluation was conducted during cold weather, when a minimum amount of fresh air exchange challenges the provision of high-quality air in the hen house. Future studies should be conducted on scenarios in which the virus is introduced from within the barn to simulate the indoor dispersal of virus particles either in a transient or steady state. In this study, virus particles were simplified as gaseous contaminants; therefore, no phase changes were considered during the numerical simulations. Although airborne or aerosolized virus particles are mostly infinitesimal and can be treated as gaseous contaminants, their transmission behavior may be expressed in a more complicated manner, such as by binding to respiratory droplets or dust particles before falling to the ground, which is worthy of further investigation. Furthermore, the broad computational domain used in this study can be employed to investigate the influence on neighboring barns and ambient environments during an outbreak event of the study hen house.

## 5. Conclusions

Numerical simulation results demonstrated that the dispersion of airborne virus particles inside a floor-raised hen house is highly dependent on internal air movements. Although all four tested models had similar ventilation rates and static pressure differences, the patterns of indoor air circulations varied with ventilation configurations, as well as the volume of indoor spaces. Simulation results of mass fractions of virus particles from bird-occupied zones at three cross-sectional reference planes suggested that the alternative ventilation designs MICE and MIAE were able to maintain up to 47% and 24% lower virus levels, respectively, on average, compared with the TISE model. The driving forces from the ceiling exhaust fan played a critical role in creating strong internal air circulations that prompted expulsion of contaminated air. In addition, the partially open attic space of the MIAE model proved beneficial to containing the spread virus particles based on the MIAE simulation results. Although the MIRE model presented virus mass fractions similar to those of the TISE model due to vigorous internal air mixing, the simulation data can be used to refine current designs.

In summary, CFD modeling is a powerful tool to investigate airborne disease spread in animal housing, allowing researchers to obtain deep insights about the relationship between ventilation configurations and disease control. The CFD models developed in this study can be further refined to evaluate other ventilation options for various types of cage-free hen houses with specific aims.

## Figures and Tables

**Table 1:** Analysis of variance (ANOVA) of mass fractions of virus particles from five bird-occupied zones at three cross-sectional reference planes (CP-I, CP-N, and CP-F) in four models.

Plane	Factor	df	Sum of Squares	Mean Square	F-Value	Pr (>F)
CP-I	Model	3	0.4	0.1	168,988	<2.2 × 10^−16^
	Zone	4	0.4	0.09	107,878	<2.2 × 10^−16^
	Model × Zone	12	0.08	0.006	7772	<2.2 × 10^−16^
	Residuals	35,320	0.03	10^−6^		
CP-N	Model	3	0.06	0.02	28,584	<2.2 × 10^−16^
	Zone	4	0.3	0.08	112,142	<2.2 × 10^−16^
	Model × Zone	12	0.03	0.003	3704	<2.2 × 10^−16^
	Residuals	34,966	0.03	10^−6^		
CP-F	Model	3	0.05	0.02	22,953	<2.2 × 10^−16^
	Zone	4	0.4	0.1	130,070	<2.2 × 10^−16^
	Model × Zone	12	0.1	0.008	11,152	<2.2 × 10^−16^
	Residuals	34,158	0.03	10^−6^		

**Table 2:** Means of mass fractions of virus particles from five bird-occupied zones at three cross-sectional reference planes (CP-I, CP-N, and CP-F) in four models. Means annotated with the same letter indicate that **NO** statistically significant differences were found between them (ANOVA and subsequent Tukey’s test, p < 0.05).

Plane	Zone	TISE	MICE	MIRE	MIAE
CP-I	Zone-1	0.0182 a	0.0122 b	0.0211	0.0171 c
	Zone-2	0.0194	0.0145	0.0223	0.0182 a
	Zone-3	0.0203	0.0068 d	0.0182 a	0.0105
	Zone-4	0.0171 c	0.0058	0.0137	0.0097
	Zone-5	0.0123 b	0.0067 d	0.0156	0.0110
CP-N	Zone-1	0.0177 a	0.0156	0.0198	0.0178 a
	Zone-2	0.0184 b	0.0168	0.0197	0.0207
	Zone-3	0.0185 b	0.0124 c	0.0152	0.0176 a
	Zone-4	0.0133	0.0098	0.0136	0.0112
	Zone-5	0.0123 c	0.0108	0.0149	0.0118
CP-F	Zone-1	0.0174	0.0220	0.0193	0.0158
	Zone-2	0.0168 a	0.0228	0.0213	0.0169 a
	Zone-3	0.0155 b	0.0138	0.0146	0.0116
	Zone-4	0.0148	0.0112 c	0.0104	0.0108
	Zone-5	0.0156 b	0.0130	0.0117	0.0113 c

## Data Availability

Not applicable.
